# *Scutellaria chenhedongensis* (Lamiaceae), a new species from Guangzhou, Guangdong, southern China

**DOI:** 10.3897/phytokeys.280.202471

**Published:** 2026-10-19

**Authors:** Xiao-Wei Yi, Yong-Fa Chen, Wei Xu, Ping-Sheng Zhong, Huan-Jin Chen, Li-Ran Shen, Qiang Fan

**Affiliations:** 1 State Key Laboratory of Biocontrol and Guangdong Provincial Key Laboratory of Plant Stress Biology, School of Life Sciences, Sun Yat-sen University, Guangzhou 510275, China State Key Laboratory of Biocontrol and Guangdong Provincial Key Laboratory of Plant Stress Biology, School of Life Sciences, Sun Yat-sen University Guangzhou China; 2 National Park and Nature Education Research Institute, Sun Yat-sen University, Guangzhou 510275, China National Park and Nature Education Research Institute, Sun Yat-sen University Guangzhou China; 3 Guangdong Conghua Chenhedong Provincial Nature Reserve, Guangzhou 510950, China Guangdong Conghua Chenhedong Provincial Nature Reserve Guangzhou China; 4 Guangdong Ecological and Environmental Monitoring Center, Guangzhou 510308, China Guangdong Ecological and Environmental Monitoring Center Guangzhou China; 5 Administrative Commission of Guangdong Nanxiong Xiaoliukeng-Qingzhangshan Provincial Nature Reserve, Shaoguan 512437, Guangdong, China Administrative Commission of Guangdong Nanxiong Xiaoliukeng-Qingzhangshan Provincial Nature Reserve Shaoguan China

**Keywords:** Lamiaceae, new species, phylogeny, *
Scutellaria
*, southern China

## Abstract

*Scutellaria chenhedongensis* X.W.Yi, Y.F.Chen & Q.Fan, a new species from Chenhedong Provincial Nature Reserve, Conghua District, Guangzhou, Guangdong Province, China, is described and illustrated. Phylogenetic analyses based on nuclear ribosomal ITS and ETS sequences resolve the three sampled individuals of the new species as a strongly supported monophyletic clade (UFBoot = 100, PP = 1.00). This clade is recovered as sister to a clade comprising *S. calcarata* and *S. jishouensis*, although this sister relationship receives weak support (UFBoot = 46, PP = 0.56). Morphologically, the new species can be clearly distinguished from these taxa by the following combination of characters: a perennial subshrubby habit with a woody stem base, reaching 40–100 cm in height; lanceolate to narrowly lanceolate leaves with conspicuously bullate venation and the abaxial surface densely covered with yellow sessile glandular dots; the co-occurrence of stipitate glandular hairs and yellow sessile glandular dots on multiple vegetative and floral organs; and a strongly geniculate corolla base lacking any saccate or spur-like projection. Morphological and molecular evidence together support its recognition as a species new to science. In accordance with IUCN Red List criteria, *S. chenhedongensis* is provisionally assessed as Vulnerable (VU D1+D2).

## Introduction

*Scutellaria* L. (Lamiaceae) is one of the most representative large genera within the family. Estimates of its species number vary considerably among sources: Paton (1990) and Harley et al. (2004) recognized approximately 360 species (Paton 1990; Harley et al. 2004; Chao et al. 2020), whereas World Flora Online currently accepts 479 species (WFO 2025). The genus has a subcosmopolitan distribution, occurring predominantly in the temperate regions of the Northern Hemisphere and in tropical montane areas, and is absent only from a few regions such as the Amazon lowlands, the lowlands of tropical Africa, and the Pacific islands (Paton 1990; Wang et al. 2024). Its center of diversity lies in the Irano-Turanian region, with species richness reaching its peak in the Caucasus and on the Iranian Plateau, while the eastern Mediterranean and the South American Andes represent important secondary centers of diversification (Paton 1990; Salimov et al. 2021; Wang et al. 2024).

Morphologically, members of the genus are mostly annual or perennial herbs, rarely subshrubs (Wu and Li 1977); the stems are quadrangular, the leaves are opposite, and the corolla is bilabiate. The most diagnostic character of the genus lies on the dorsal side of the upper calyx lip, where an erect, sail- or shield-shaped appendage—termed the scutellum—is borne, and from which the generic name *Scutellaria* is derived. In addition, a saccate or spur-like projection at the base of the corolla tube is also an important identifying feature of the genus (Paton 1990; Harley et al. 2004; Chao et al. 2020).

As a major group of traditional medicinal plants, *Scutellaria* occupies a distinctive position in East Asian medicine. The roots of *S. baicalensis* Georgi have been used medicinally for over two millennia and, together with several closely related species such as *S. barbata* D.Don and *S. indica* L., constitute a widely employed group of “heat-clearing and detoxifying” (qing-re jie-du) materia medica in traditional Chinese medicine (Shang et al. 2010; Wang et al. 2018; Wang et al. 2024). Modern pharmacological studies have shown that these species are rich in flavonoids and exhibit a range of bioactivities, including antitumor, anti-inflammatory, antioxidant, and hepatoprotective effects (Miyaichi et al. 1987, 1989; Sato et al. 2000; Shang et al. 2010; Kudo et al. 2017; Wang et al. 2018). The continued discovery of their medicinal value has further stimulated interest in the species diversity of the genus.

China is the main area of distribution for *Scutellaria* in East Asia. According to the “Flora of China”, the genus is represented in the country by approximately 100 species and 25 varieties (Li and Hedge 1994; Xiang 2016), concentrated mainly in the mountains of southwestern China and the hilly regions of southern China. However, because interspecific morphological differences within the genus are often subtle and many closely related species exhibit continuous variation in the field, the interspecific relationships of Chinese *Scutellaria* as recognized under traditional morphology-based classification remain insufficiently resolved (Wang et al. 2024). Over the past decade, with the widespread application of nuclear ribosomal markers such as ITS and ETS and the incorporation of plastome data, considerable progress has been made in species delimitation and phylogenetic placement of Chinese *Scutellaria*, and a number of new taxa have been described in succession: *S. wuana* C.L.Xiang & F.Zhao from Sichuan (Zhao et al. 2017), *S. jishouensis* G.X.Chen, L.Tan & X.M.Xiang from Hunan (Tan et al. 2022), *S. wushanensis* H.P.Deng & H.Zhang from Chongqing (Zhang et al. 2024), *S. cavicola* C.Xiong, Z.Y.Wei & L.F.Fu from a karst cave entrance in Guangxi (Xiong et al. 2025), and *S. albovillosa* Y.P.Zeng and *S. yangchunensis* Y.P.Zeng, both described from southwestern Guangdong (Zeng et al. 2025). Notably, three new species were described from southern China in 2025 alone (Xiong et al. 2025; Zeng et al. 2025), suggesting that further taxonomic investigation of *Scutellaria* in this region is warranted.

In March 2026, during a floristic survey at Chenhedong Provincial Nature Reserve in Conghua District, Guangzhou, Guangdong Province, a morphologically distinctive species of *Scutellaria* was collected along a forest-edge valley. The plant exhibited a subshrubby habit with a woody stem base, a secund terminal raceme, and a strongly geniculate corolla base lacking any saccate or spur-like projection. After consulting the relevant literature and herbarium specimens, the plant could not be assigned to any known species. Molecular phylogenetic analyses based on nuclear ribosomal ITS and ETS sequences further revealed that this plant is sister to *S. calcarata* C.Y.Wu & H.W.Li from Yunnan and *S. jishouensis* G.X.Chen, L.Tan & X.M.Xiang from Hunan, but differs consistently from both in corolla base structure, inflorescence morphology, and vegetative characters. Based on the combined morphological and molecular evidence, the plant is here formally described as a new species of *Scutellaria*, and its morphology, phylogenetic placement, habitat, and conservation status are presented.

## Materials and methods

### Morphological study

Specimens of the new species were collected during a field expedition to Chenhedong Provincial Nature Reserve in Conghua District, Guangzhou, Guangdong Province, on 28 March 2026. Field photographs were taken using a Sony α7C II digital camera. All morphological characters were examined on both field-collected material and herbarium specimens. Close-up images of floral structures and nutlets were captured with the same camera fitted with a macro lens, while details of indumentum and glandular dots were photographed using a ZEISS Axiocam 208 color camera mounted on a ZEISS Stemi 305 stereomicroscope. Voucher specimens were deposited at the Herbarium of Sun Yat-sen University (SYS). Digitized specimens of closely related species were examined through the Chinese Virtual Herbarium (https://www.cvh.ac.cn/) and the Global Biodiversity Information Facility (https://www.gbif.org/) to obtain morphological data for comparison. Terminology for morphological descriptions follows the “Flora of China” (Li and Hedge 1994).

### Molecular phylogenetic study

Fresh leaves of three individuals of the new species were collected at the type locality in Chenhedong Provincial Nature Reserve and preserved in silica gel. Total genomic DNA was extracted using a modified CTAB method (Doyle and Doyle 1987). The nuclear ribosomal internal transcribed spacer (ITS) region was amplified by PCR using the universal primers ITS1/ITS4 (White et al. 1990), and the external transcribed spacer (ETS) region was amplified using the primers ETS-B (Beardsley and Olmstead 2002) and 18S-IGS (Baldwin and Markos 1998). PCR amplifications followed standard protocols with an annealing temperature of 55 °C for both ITS and ETS. PCR products were sent to Tianyi Huiyuan Bioscience & Technology Co., Ltd. (Guangzhou, China) for bidirectional Sanger sequencing. The newly generated ITS and ETS sequences of the new species have been deposited in GenBank.

To determine the phylogenetic position of the new species, the sampling strategy of Xiong et al. (2025) was followed, and ITS and ETS sequences of 43 *Scutellaria* taxa were downloaded from GenBank, comprising 44 ITS sequences and 43 ETS sequences in total (Xiong et al. 2025). Sequences of *S. cavicola* C.Xiong, Z.Y.Wei & L.F.Fu and *S. tsinyunensis* C.Y.Wu & S.Chow were not included, as they had not yet been publicly released in GenBank at the time of analysis. The final dataset comprised 52 accessions, representing 46 taxa: 43 species and one variety of *Scutellaria* as the ingroup (including three individuals of the new species), with one species each of *Holmskioldia* Retz. and *Tinnea* Kotschy ex Hook.f. as outgroups. Voucher information and GenBank accession numbers for all sequences used in the analyses are listed in Table 1.

**Table 1.** Voucher information and GenBank accession numbers for taxa included in the phylogenetic analyses. Sequences newly generated in this study are indicated in bold.

**Taxa**	**Voucher/Herbarium barcode**	**Location**	**ITS**	**ETS**
*S. alpina*	Liao PC, s.n.	Europe alpine region	MF193544	MF193591
*S. axilliflora*	Hu GX, H144 (KUN)	Fujian, China	MF193529	MF193575
*S. baicalensis*	Li DZ et al., 0513 (KUN)	Liaoning, China	MF193525	MF193571
*S. barbata*	Xiang CL, 282 (KUN)	Beijing, China	MF193539	MF193585
*S. calcarata*	Shui YM et al., Z-03343396 (KUN)	Yunnan, China	MF193512	MF193558
*S. dependens*	Anonymous, 316	Fujinomiya, Japan	MF193538	MF193584
*S. diffusa*	Wang ZH, s.n. (KUN)	Germany	MF193541	MF193587
*S. discolor*	Xiang CL et al., 438 (KUN)	Yunnan, China	MF193504	MF193550
*S. franchetiana*	Xiang CL, 287 (KUN)	Yunnan, China	MF193532	MF193578
*S. galericulata*	M-14212	Iran	MF193535	MF193581
*S. hainanensis*	Jiang L et al., 398 (KUN)	Hainan, China	MF193505	MF193551
*S. hunanensis*	Hu GX, H96 (KUN)	Hunan, China	MF193531	MF193577
*S. indica*	Peng H, s.n. (KUN)	Hong Kong, China	MF193513	MF193559
*S. jishouensis*	Chen GX et al., DHK-20210418	Hunan, China	OM287561	OM307410
*S. kingiana*	Zhang JW et al., ZJW-3890 (KUN)	Xizang, China	MF193542	MF193588
*S. likiangensis*	Xiang CL et al., 373 (KUN)	Yunnan, China	MF193524	MF193570
*S. macrodonta*	Zhao F et al., 2015-006 (KUN)	Beijing, China	MF193523	MF193569
*S. mairei*	Shui YM et al., 66205 (KUN)	Yunnan, China	MF193516	MF193562
*S. nepetifolia*	TUH-27605 (THU)	Iran	MF193545	MF193592
*S. nuristanica*	M-32142	Iran	–	MF193589
*S. obtusifolia*	Chen YP et al., EM202 (KUN)	Sichuan, China	MF193508	MF193554
*S. orthocalyx*	Xiang CL, 185 (KUN)	Yunnan, China	MF193527	MF193573
*S. platystegia*	TUH-7697 (THU)	Iran	MF193546	MF193593
*S. regeliana*	Jiang L, 149 (KUN)	Neimenggu, China	MF193536	MF193582
*S. scordiifolia*	Yu WT et al., 2822 (KUN)	Qinghai, China	MF193540	MF193586
*S. sessilifolia* 1	Xiang CL, 341 (KUN)	Chongqing, China	MF193533	MF193579
*S. sessilifolia* 2	Peng H et al., 117 (KUN)	Sichuan, China	MF193534	MF193580
*S. shweliensis*	Zhao F et al., ZF0068 (KUN)	Yunnan, China	MF193530	MF193576
*S. sichourensis*	Xiang CL et al., 566 (KUN)	Yunnan, China	MF193509	MF193555
*S. stocksii*	M-30348	Iran	MF193543	MF193590
*S. subintegra* 1	Chen YP, EM223 (KUN)	Guangxi, China	MF193528	MF193574
*S. supina*	Liu B et al., CPG28095 (PE)	Xinjiang, China	MF193547	MF193594
*S. taiwanensis*	Liao PC, s.n. (KUN)	Taiwan, China	MF193515	MF193561
*S. tapintzensis*	Cai J et al., 15cs11358 (KUN)	Yunnan, China	MF193518	MF193564
*S. tenax*	Peng H et al., 2012-017 (KUN)	Guizhou, China	MF193517	MF193563
*S. tenera*	Chen YP et al., EM187 (KUN)	Jiangxi, China	MF193522	MF193568
*S. teniana*	Xiang CL et al., 288 (KUN)	Yunnan, China	MF193520	MF193566
*S. viscidula*	Zhao F, 2015-009 (KUN)	Hebei, China	MF193526	MF193572
*S. wenshanensis*	Zhao F et al., 008 (KUN)	Yunnan, China	MF193510	MF193556
*S. wongkei* 1	–	Guangdong, China	PQ565175	–
*S. wongkei* 2	–	Guangdong, China	PQ565021	–
*S. wuana*	Xiang CL et al., 1200 (KUN)	Sichuan, China	MF193521	MF193567
*S. yangbiensis*	Liu ED et al., 2238 (KUN)	Yunnan, China	MF193511	MF193557
S. yunnanensis var. cuneata	Xiang CL et al., 574 (KUN)	Yunnan, China	MF193507	MF193553
*S. yunnanensis*	Liu ED et al., 3037 (KUN)	Yunnan, China	MF193506	MF193552
***S. chenhedongensis*** 1	**HQ-1001-1 (SYS)**	**Guangdong, China**	**PZ492709**	**PZ493507**
***S. chenhedongensis*** 2	**HQ-1001-2 (SYS)**	**Guangdong, China**	**PZ492710**	**PZ493508**
***S. chenhedongensis*** 3	**HQ-1001-3 (SYS)**	**Guangdong, China**	**PZ492711**	**PZ493509**
***S. subintegra*** 2	–	**Guangdong, China**	–	–
***S. subintegra*** 3	–	**Guangdong, China**	–	–
*Holmskioldia sanguinea*	Anonymous, 209	Guangdong, China	MF193548	MF193595
*Tinnea rhodesiana*	Gary Stafford, GIS-359 (KUN)	South Africa	MF193549	MF193596

The ITS and ETS sequences were aligned separately using MAFFT v. 7 with the options --auto --adjustdirection (Katoh and Standley 2013). Poorly aligned regions were trimmed using trimAl v. 1.4 with the -automated1 option (Capella-Gutiérrez et al. 2009). After trimming, the ITS and ETS alignments were concatenated for subsequent phylogenetic analyses.

Maximum likelihood (ML) analysis was performed on the partitioned concatenated ITS+ETS matrix in IQ-TREE v. 2 (Minh et al. 2020). The best-fitting substitution model for each partition was selected using the built-in ModelFinder (Kalyaanamoorthy et al. 2017) under the Bayesian information criterion: GTR+F+I+G4 for ITS and TVM+F+I+R2 for ETS. Branch support was assessed using 3,000 ultrafast bootstrap (UFBoot) replicates (Hoang et al. 2018) and 1,000 SH-aLRT replicates (Guindon et al. 2010).

For Bayesian inference (BI), ITS and ETS were treated as separate partitions in MrBayes v. 3.2.7 (Ronquist et al. 2012). Because the partition-specific models selected in IQ-TREE are not implemented exactly in MrBayes, both partitions were assigned the MrBayes-compatible GTR+I+G model. Substitution model parameters, including base frequencies, exchangeability rates, gamma-shape parameters, and proportions of invariant sites, were estimated independently for each partition, and partition-specific rate multipliers were allowed to vary. Two independent runs were performed, each with four Markov chains (one cold and three heated; temp = 0.1) for 10,000,000 generations, sampling every 1,000 generations and discarding the first 25% as burn-in. Convergence was assessed using the average standard deviation of split frequencies (ASDSF < 0.01) and effective sample sizes (ESS > 200) for all parameters. The resulting phylogenetic tree was visualized using FigTree v. 1.4.4 (Rambaut 2018).

## Results and discussion

### Molecular phylogenetic analysis

**Figure 1.**
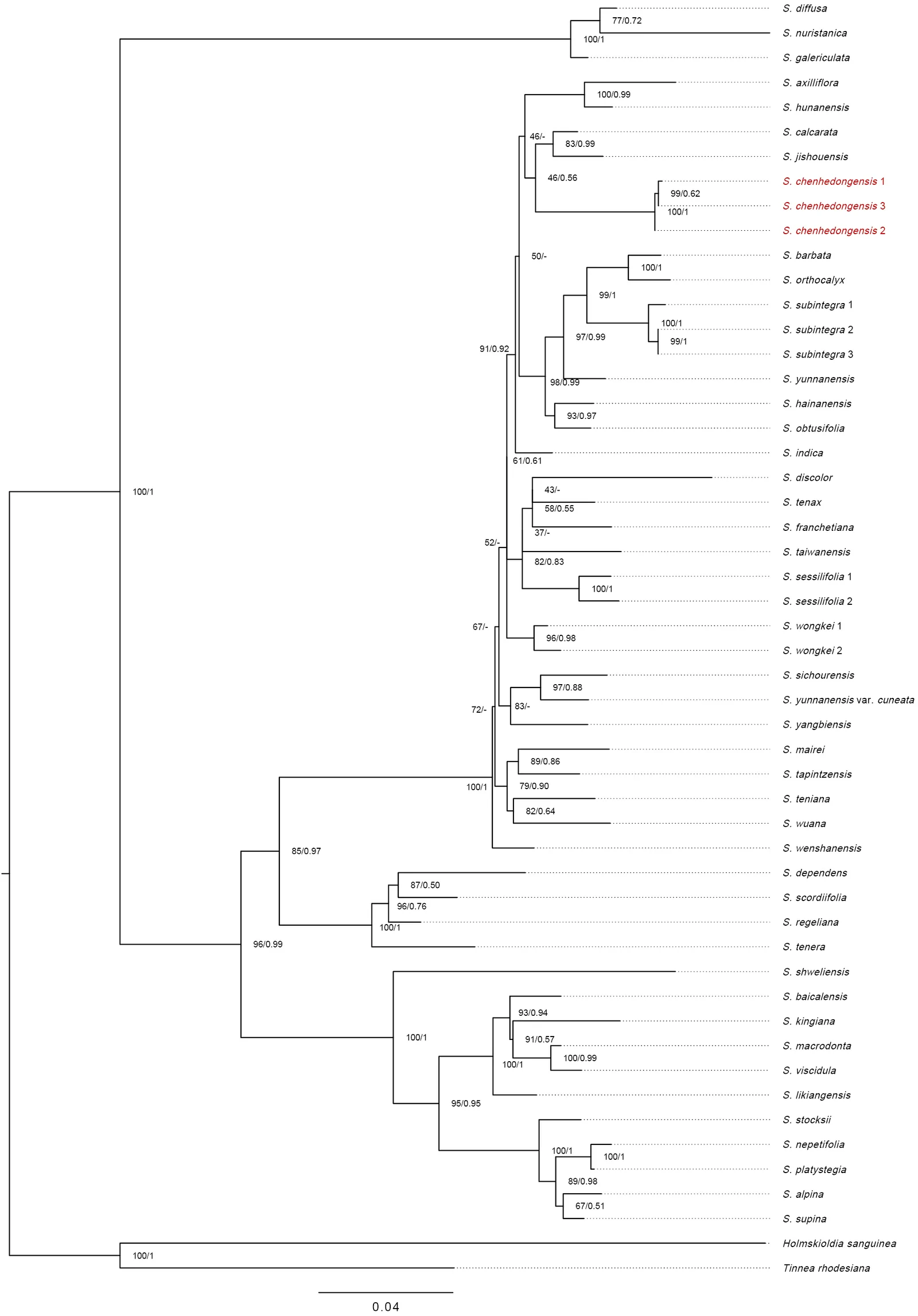
Maximum likelihood tree inferred from combined ITS and ETS sequence data. Numbers at nodes indicate ultrafast bootstrap support/Bayesian posterior probabilities; “-” denotes nodes not resolved in the Bayesian analysis or with PP < 0.5. The new species is highlighted in red.

After MAFFT alignment and trimAl trimming, the ITS and ETS alignments were 598 bp and 394 bp in length, respectively, and were concatenated into a matrix of 992 bp. The topologies recovered by ML and BI analyses were highly congruent; therefore, only the ML tree is shown (Fig. 1), with UFBoot values and BI posterior probabilities (PP) annotated at each node.

The phylogenetic results are largely consistent with previous studies (Tan et al. 2022; Xiong et al. 2025). The three individuals of *S. chenhedongensis* sampled in this study formed a strongly supported monophyletic clade (UFBoot = 100, PP = 1.00). This clade was recovered as sister to a clade comprising *S. calcarata* and *S. jishouensis*, which was strongly supported (UFBoot = 83, PP = 0.99); however, the sister relationship between *S. chenhedongensis* and the *S. calcarata*–*S. jishouensis* clade received weak support (UFBoot = 46, PP = 0.56) (Fig. 1). *Scutellaria axilliflora* and *S. hunanensis* also formed a strongly supported clade (UFBoot = 100, PP = 0.99), whereas its relationship with the above lineages was weakly supported in the ML analysis and unresolved in the BI consensus tree. Therefore, relationships among these closely related lineages should be interpreted with caution and require further evidence from broader sampling and additional molecular data.

The phylogenetic placement of *S. chenhedongensis* also highlights incongruence between traditional morphology-based infrageneric classification and molecular evidence. Under the classification of Wu and Li (1977), *S. calcarata* was assigned to Ser. *Violaceae*, whereas *S. axilliflora* and *S. hunanensis* were assigned to Ser. *Axilliflorae*; *S. jishouensis*, described in 2022, has not yet been formally assigned to a series (Wu and Li 1977; Tan et al. 2022). The recovery of these lineages in the present analyses is consistent with the phylogenomic findings of Wang et al. (2024), which indicated that the traditional infrageneric classification of *Scutellaria* is largely incongruent with molecular evidence.

Although the sister relationship between *S. chenhedongensis* and the clade comprising *S. calcarata* and *S. jishouensis* received only weak support (UFBoot = 46, PP = 0.56), the monophyly of *S. chenhedongensis* itself was recovered with the highest possible support in all analyses (UFBoot = 100, PP = 1.00). Combined with the stable morphological differences detailed below, this strongly supports its recognition as a distinct species.

### Morphological comparison

Based on phylogenetic placement and morphological similarity, *S. chenhedongensis* is compared in detail with the four most closely related species within the same clade: *S. calcarata*, *S. jishouensis*, *S. hunanensis*, and *S. axilliflora* (Table 2).

**Table 2.** Morphological comparison between *Scutellaria chenhedongensis* and its closely related congeners.

**Character**	***S. chenhedongensis***	***S. calcarata***	***S. jishouensis***	***S. hunanensis***	***S. axilliflora***
Habit	Perennial subshrub	Annual herb	Perennial herb	Perennial herb*	Perennial herb
Plant height (cm)	40–100	≤ 30	2–4	20–40*	30–40 (60)
Stem indumentum	Densely strigose	Densely puberulent	Sparsely puberulent	Antrorsely appressed-puberulent	Antrorsely curved-puberulent
Leaf shape	Lanceolate to narrowly lanceolate	Cordate	Ovate to orbicular	Triangular-ovate	Ovate to triangular-ovate
Leaf size (cm)	1.5–5.2 × 0.5–1.5	2.3–4 × 1.8–3.5	0.5–2.9 × 0.6–3	2.5–5.5 × 1.5–4	1.2–2.5 × 0.7–2.1
Leaf texture and surface	Strigose, bullate	Sparsely strigose	Glabrous, membranous	Adaxially jointed-strigose	Sparsely puberulent
Leaf glandular dots	Abaxially dense	Absent	Absent	Adaxially present	On both surfaces
Inflorescence type	Dense terminal raceme	Lax terminal raceme	Lax terminal raceme	Lax terminal raceme	Flowers solitary in bracteal axils
Rachis indumentum	Densely glandular-pubescent, punctate	Densely glandular-puberulent	Sparsely puberulent	Puberulent	Puberulent
Calyx indumentum	Densely glandular-pubescent, punctate	Puberulent	Sparsely pilose	Puberulent, punctate	Puberulent, punctate
Corolla length (cm)	1.8–2.4	ca. 2	1.0–1.8	0.9–1.3*	1.6–2
Corolla color	Pale to pinkish-purple	Magenta	Bluish-purple	White*	Purple to pale bluish-purple
Corolla indumentum	Densely glandular-pubescent, sparsely punctate distally	Densely glandular-puberulent	Sparsely puberulent	–	Sparsely puberulent
Corolla base	Strongly geniculate, exappendiculate	Geniculate, with a saccate spur ca. 2.5 mm	Straight, with a saccate projection	Geniculate, exappendiculate*	Geniculate, exappendiculate
Type locality	Guangzhou, Guangdong	Dayao, Yunnan	Jishou, Hunan	Nanyue, Hunan	Yanping, Fujian
Habitat	Valley margins	Montane streamsides	Damp cliffs	Forest understorey	Shaded rocks
Elevation (m)	444	ca. 2,650	300–700	–	ca. 900
Flowering period	March–April	September	April–June	May*	April–June

“-” indicates data not available from the original description; asterisks (*) indicate data inferred from specimen records and photographs rather than directly stated in the protologue.

*Scutellaria chenhedongensis* can be unambiguously distinguished from all four congeners by a combination of diagnostic characters. The most diagnostic feature is its perennial subshrubby habit, which is unique within the clade; the other four species are herbaceous (*S. calcarata* being annual and the remaining species perennial). In terms of plant height, *S. chenhedongensis* reaches 40–100 cm. The other four species generally do not exceed 40 cm, although *S. axilliflora* occasionally reaches 60 cm. Its lanceolate to narrowly lanceolate leaves contrast sharply with the cordate leaves of *S. calcarata*, the ovate to orbicular leaves of *S. jishouensis*, and the triangular-ovate leaves of *S. hunanensis* and *S. axilliflora*.

A second core diagnostic feature of *S. chenhedongensis* lies in the combined distribution pattern of glandular hairs and yellow glandular dots. The rachis, pedicels, and outer surface of the calyx are all densely glandular-pubescent and densely yellow-punctate; the outer surface of the corolla is also densely glandular-pubescent but bears only sparsely scattered yellow glandular dots, mainly toward the distal portion; in addition, the stem bears scattered yellow glandular dots, and the abaxial leaf surface is densely yellow-punctate. The co-occurrence of glandular hairs and glandular dots on multiple organs is unique to *S. chenhedongensis* among the five species compared. *S. calcarata* possesses glandular puberulence on the floral parts, but discrete yellow glandular dots have not been reported; *S. hunanensis* and *S. axilliflora* have been described as bearing glandular dots only on the leaf surface or calyx, with no glandular hairs recorded; and *S. jishouensis* has been described as bearing only sparse non-glandular pilosity, with neither glandular hairs nor glandular dots reported (Tan et al. 2022).

The structure of the corolla base provides the clearest distinction from the closest comparative taxon, *S. calcarata*. The corolla tube of *S. chenhedongensis* is strongly geniculate at the base but lacks any saccate or spur-like projection, whereas that of *S. calcarata* bears a saccate spur ca. 2.5 mm long projecting forward from the base (Wu and Li 1977), the very feature from which the specific epithet “calcarata” is derived. The distinction from *S. jishouensis* is even more pronounced: the latter has a straight corolla tube with a saccate projection in front (Tan et al. 2022), and, in combination with its creeping habit (stems only 2–4 cm tall) and membranous, wholly glabrous leaves, the two species are readily distinguishable in the field. The distinction from *S. axilliflora* lies chiefly in the inflorescence type: *S. chenhedongensis* bears a dense, secund, terminal raceme, whereas in *S. axilliflora*, the flowers are solitary in the axils of bracteal leaves rather than aggregated into a raceme.

The five species occur in different parts of southern to southwestern China: *S. chenhedongensis* in Guangzhou, Guangdong (444 m); *S. calcarata* in Dayao, Yunnan (ca. 2,650 m); *S. jishouensis* in Jishou, Hunan (300–700 m); *S. hunanensis* in Nanyue, Hunan; and *S. axilliflora* in Yanping, Fujian. *Scutellaria chenhedongensis* and its closest relative, *S. calcarata*, are completely allopatric, separated by over 1,200 km in linear distance and over 2,200 m in elevation, and their flowering periods are offset by nearly half a year (*S. chenhedongensis* in March–April vs. *S. calcarata* in September). Although the molecular evidence indicates a close phylogenetic relationship between the two species, the geographic and phenological separation between them constitutes a strong reproductive barrier.

### Life-history variation among the taxa compared

Among the taxa compared here, *S. chenhedongensis*, *S. calcarata*, *S. jishouensis*, *S. hunanensis*, and *S. axilliflora* display marked variation in growth form and stature. *Scutellaria chenhedongensis* is a perennial subshrub with a woody stem base, reaching up to 1 m in height; *S. calcarata* is an annual herb only ca. 30 cm tall; *S. jishouensis* is a diminutive perennial herb with creeping stems only a few centimeters long and adventitious roots; and *S. hunanensis* and *S. axilliflora* are erect perennial herbs. The contrast between *S. chenhedongensis* and *S. jishouensis* is particularly pronounced: the former is tall and woody-based, approaching a subshrubby form, whereas the latter is diminutive, wholly herbaceous, and creeping on cliff faces. These differences illustrate substantial variation in life-history characters among the morphologically and phylogenetically associated taxa examined in this study.

In addition, the subshrubby habit with a woody stem base also occurs in *S. tsinyunensis* (from Jinyun Mountain, Chongqing) and the recently described *S. cavicola* (from the entrance of a karst cave in Lingyun County, Guangxi) (Xiong et al. 2025). According to the phylogenetic analysis of Xiong et al. (2025), *S. tsinyunensis* and *S. cavicola* are sister to each other and are more closely related to *S. indica*. This sister pair is not directly adjacent to the smaller clade containing *S. chenhedongensis*. This implies that the subshrubby habit may have arisen independently at least twice within this larger clade. Given that all three subshrubby species grow in moist, shaded subtropical habitats at low to mid elevations (either karst cave entrances or valleys within evergreen broad-leaved forests), the lignification of the stem base may represent a convergent adaptation to similar environmental pressures.

These results indicate that, even within closely related clades, life-history characters in *Scutellaria* may undergo considerable divergence and convergence. This pattern provides a valuable system for future investigations into the evolutionary mechanisms and ecological drivers underlying life-history variation in the genus.

### Taxonomic treatment

#### Scutellaria chenhedongensis

Taxon classification

Plantae

LamialesLamiaceae

X.W.Yi, Y.F.Chen & Q.Fan sp. nov.

3A9B1E23-AB1E-5D99-9705-068AAEAA9AE2

urn:lsid:ipni.org:names:77413063-1

Figs 2, 3, 4, 5, 6

##### Chinese name.

陈禾洞黄芩 (chén hé dòng huáng qín).

**Figure 2.**
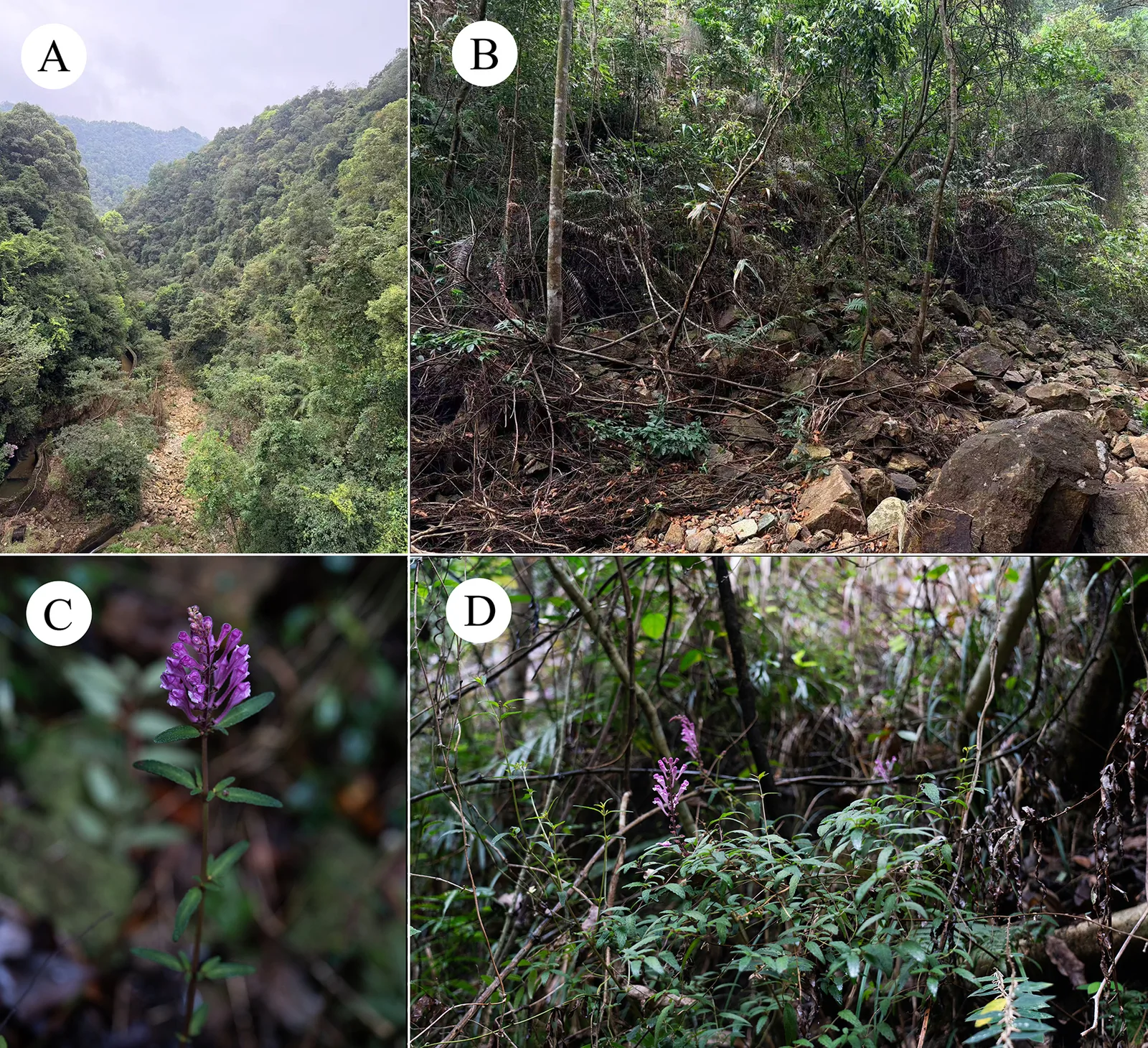
*Scutellaria chenhedongensis* in its natural habitat. **A, B**. Habitat at the type locality, Chenhedong Provincial Nature Reserve, Conghua, Guangdong; **C, D**. Flowering plants *in situ*.

**Figure 3.**
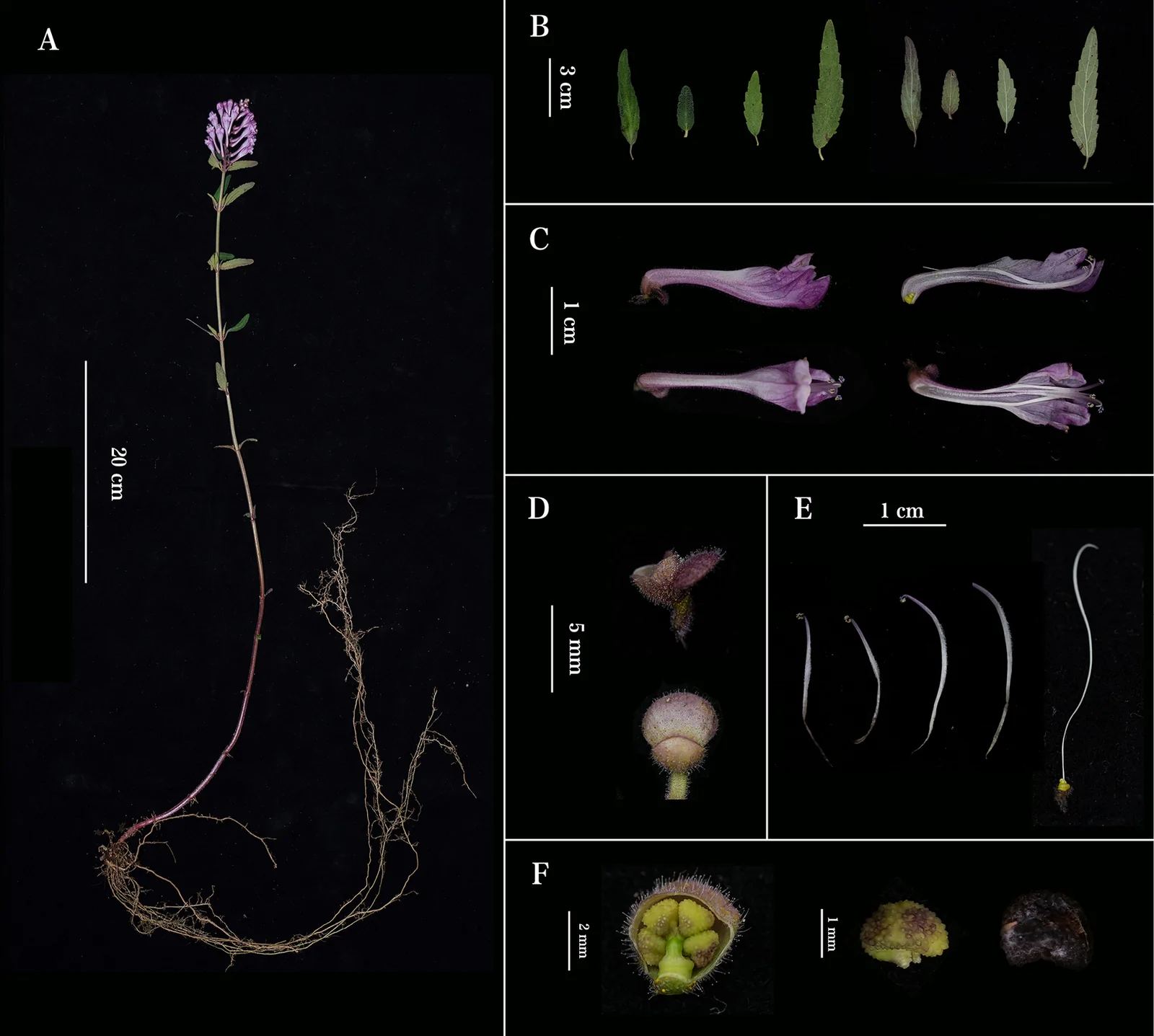
Morphology of *Scutellaria chenhedongensis*. **A**. Whole plant showing the woody stem base and fibrous root system; **B**. Leaves, with the adaxial surface on the left and the abaxial surface on the right; **C**. Flower in lateral view (upper left), frontal view (lower left), and longitudinal section (right); **D**. Calyx with scutellum in lateral view (upper) and frontal view (lower); **E**. Stamens and gynoecium; **F**. Fruiting calyx in longitudinal section showing immature nutlets (left), immature nutlet (middle), and mature nutlet (right).

**Figure 4.**
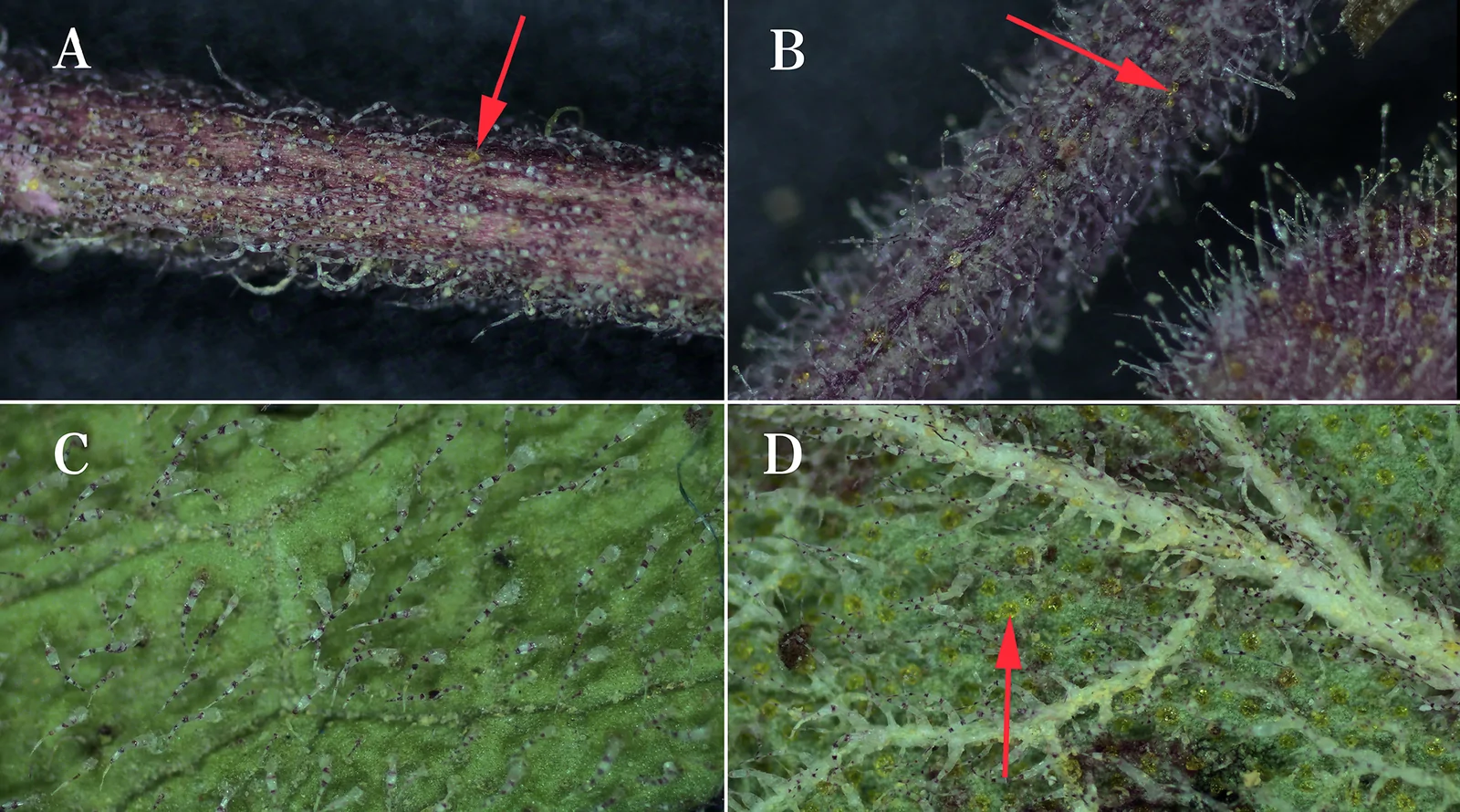
Indumentum and glandular features of *Scutellaria chenhedongensis* observed under a stereomicroscope. **A**. Stem surface showing multicellular strigose hairs and scattered yellow sessile glandular dots (arrows); **B**. Rachis showing dense stipitate glandular hairs and yellow sessile glandular dots (arrows); **C**. Adaxial leaf surface showing multicellular strigose hairs without visible glandular dots; **D**. Abaxial leaf surface showing multicellular strigose hairs and dense yellow sessile glandular dots (arrows).

**Figure 5.**
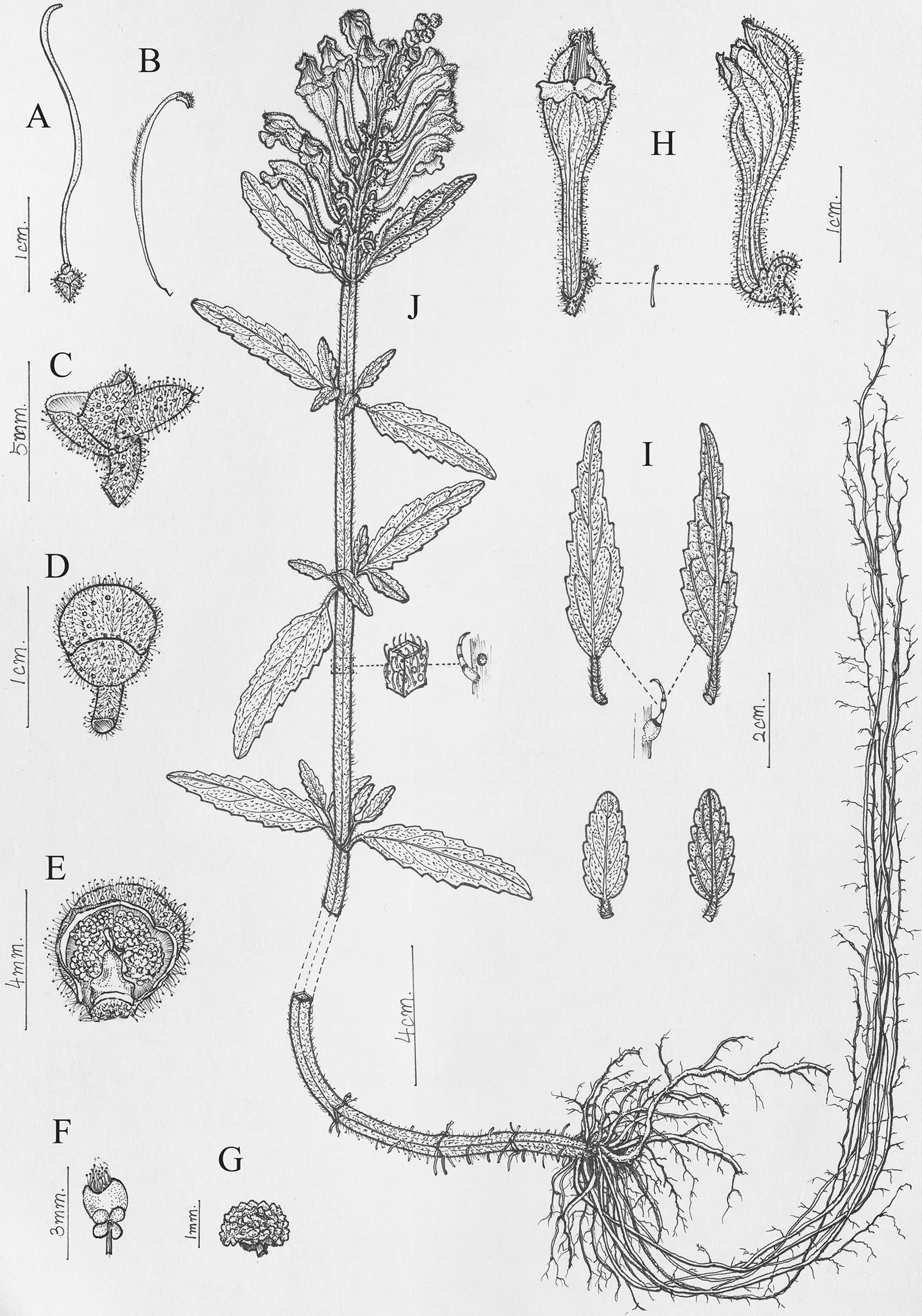
Botanical illustration of *Scutellaria chenhedongensis*. **A**. Gynoecium; **B**. Stamen; **C**. Calyx; **D**. Fruiting calyx; **E**. Longitudinal section of the fruiting calyx, showing four nutlets; **F**. Dissected ovary at anthesis, showing four ovules; **G**. Mature nutlet; **H**. Corolla in lateral view (left) and longitudinal section (right), with an enlarged view of a stipitate glandular hair; **I**. Leaves, showing both surfaces and an enlarged view of multicellular strigose hairs; **J**. Habit, with enlarged details of multicellular strigose hairs and yellow sessile glandular dots on the stem. Illustration by Rong-En Wu.

**Figure 6.**
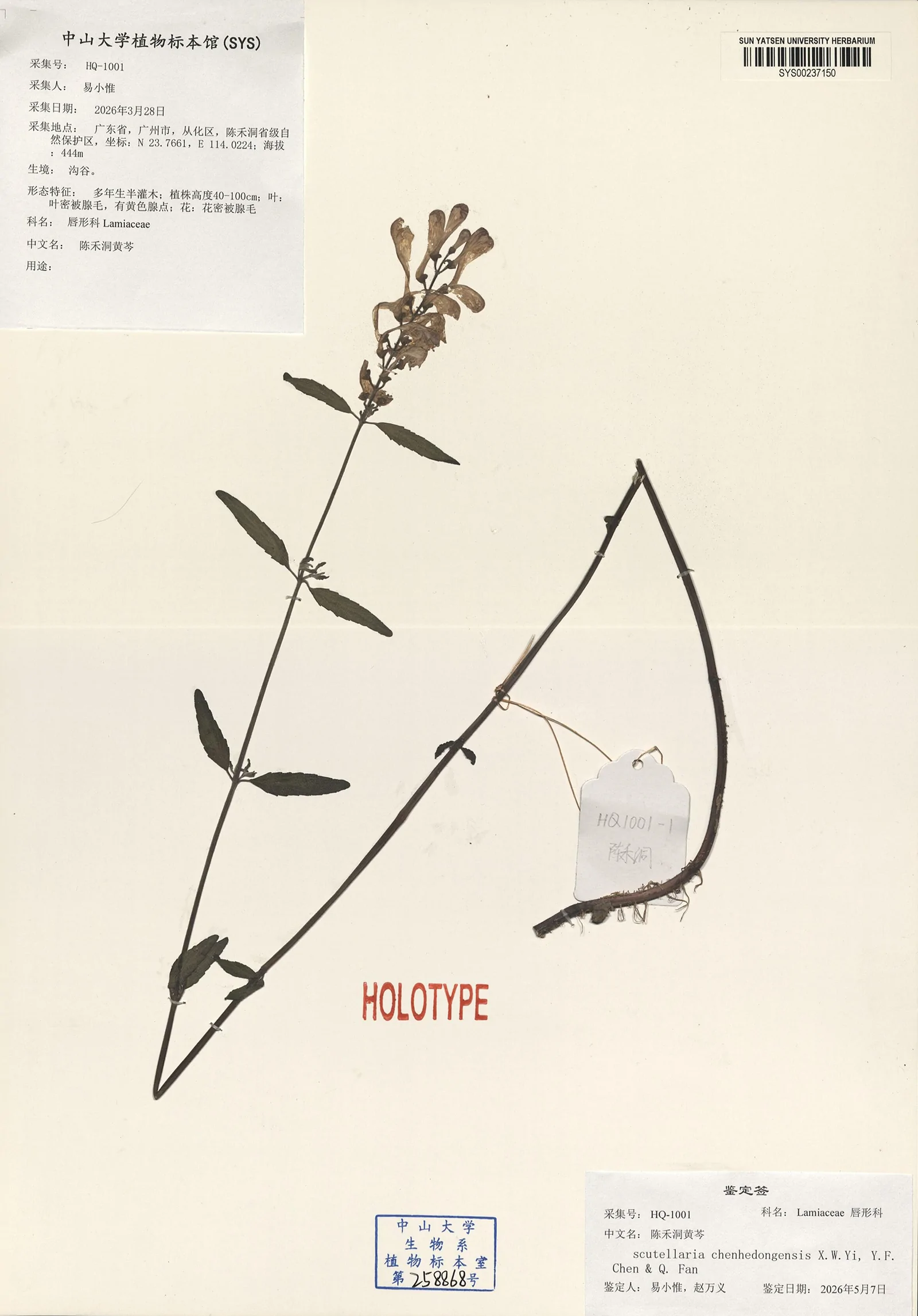
Holotype specimen of *Scutellaria chenhedongensis* X.W.Yi, Y.F.Chen & Q.Fan (HQ-1001; SYS00237150, SYS).

##### Type.

China • Guangdong Province: Guangzhou City, Conghua District, Chenhedong Provincial Nature Reserve, alt. 444 m, 23.766077, 114.022436 (WGS84), 28 March 2026, *X.W. Yi HQ-1001* (holotype: SYS [SYS00237150]).

##### Diagnosis.

*Scutellaria chenhedongensis* is distinguished from all other species of *Scutellaria* by the following combination of characters: a perennial subshrubby habit with a woody stem base and plants 40–100 cm tall; the co-occurrence of stipitate glandular hairs and yellow sessile glandular dots distributed across multiple vegetative and floral organs; and a strongly geniculate corolla base lacking any saccate or spur-like projection.

Phylogenetically, *S. chenhedongensis* is placed near *S. calcarata* C.Y.Wu & H.W.Li (from Yunnan) and *S. jishouensis* G.X.Chen, L.Tan & X.M.Xiang (from Hunan), but can be unambiguously distinguished from both by a combination of stable morphological characters.

From *S. calcarata* it differs in being a perennial subshrub 40–100 cm tall with a woody stem base (vs. an annual herb up to 30 cm tall); in having leaves lanceolate to narrowly lanceolate, 1.5–5.2 cm long (vs. cordate, 2.3–4 cm long); in the corolla base being strongly geniculate and exappendiculate, lacking any saccate or spur-like projection (vs. bearing a saccate spur ca. 2.5 mm long projecting forwards from the base); and in its flowering period of March-April (vs. September).

From *S. jishouensis* it differs in having erect, subshrubby stems 40–100 cm tall (vs. creeping to ascending-erect stems only 2–4 cm tall, bearing adventitious roots); in its chartaceous leaves strigose on both surfaces, with the abaxial surface densely yellow-punctate (vs. membranous to slightly fleshy leaves, glabrous on both surfaces and eglandular); in having the abaxial leaf surface grayish-green (vs. purple); and in the corolla base being strongly geniculate and lacking any saccate projection (vs. a straight corolla tube with a saccate projection in front).

A detailed morphological comparison is provided in Table 2.

##### Description.

***Perennial subshrub***, 40–100 cm tall, with a woody stem base; fibrous roots well developed, arising from a short rhizome. ***Stems*** erect, quadrangular, branched at the base and much-branched above; green throughout, gradually turning purplish-red towards the base; densely covered with multicellular strigose hairs, becoming sparser towards the base, and sparsely bearing yellow sessile glandular dots.

***Leaves*** opposite, chartaceous; petioles 3–10 mm long, densely strigose; leaf blades lanceolate to narrowly lanceolate, rarely ovate, 1.5–5.2 × 0.5–1.5 cm; base cuneate, gradually narrowing and shortly decurrent onto the petiole; apex acute to obtuse; margin obtusely crenate; adaxial surface dark green, with the reticulate venation deeply impressed and conspicuously bullate, strigose, eglandular; abaxial surface grayish-green, contrasting markedly in color with the adaxial surface, strigose, densely covered with yellow sessile glandular dots; lateral veins in 5–8 pairs, prominently raised abaxially.

***Inflorescence*** a terminal, secund, dense raceme, 3.5–8 cm long; flowers densely arranged with very short pedicels, giving the inflorescence a nearly spicate appearance; rachis densely covered with stipitate glandular hairs and dense yellow sessile glandular dots. Pedicels 1.5–3 mm long, densely covered with stipitate glandular hairs and dense yellow sessile glandular dots.

***Calyx*** bilabiate, ca. 1.5–2 mm long, shortly tubular; outer surface densely covered with stipitate glandular hairs and dense yellow sessile glandular dots, the glandular heads conspicuous; upper lip bearing an erect, low, shield-shaped scutellum; calyx purplish-red, concolorous with the rachis.

***Corolla*** 1.8–2.4 cm long, pale purple to pinkish-purple, the lower lip marked with deep purple veins, the throat and inner surface of the lower lip paler; outer surface densely covered with stipitate glandular hairs, the hairs long and conspicuous; sparsely bearing yellow sessile glandular dots towards the distal portion of the outer surface. Corolla tube strongly geniculate at the base but lacking any saccate or spur-like projection; tube slender, narrow at the base, slightly constricted in the middle, and strongly dilated at the throat. Upper lip galeate, strongly arched. Lower lip three-lobed; median lobe markedly larger than the lateral lobes; lower lip strongly reflexed downwards and outwards. Stamens four, didynamous, concealed beneath the galea. Ovary 4-lobed; style gynobasic.

***Fruiting calyx*** strongly enlarged and closed, ca. 4–5 × 3.5–4 mm; scutellum hemispherical to broadly reniform, conspicuously enlarged at maturity. Nutlets four, free, arranged in the typical cruciate-symmetrical pattern; subhemispherical to broadly ovoid, ca. 1.0–1.2 mm in diameter; ventral surface flat, dorsal surface convex; mature nutlets deep black with a faint lustre, densely covered with conical to hemispherical papillae of uniform size; hilum lateral at the base. Immature nutlets pale yellowish-green, with papillae already beginning to develop.

##### Phenology.

Flowering from March to April; fruiting from April to May.

##### Etymology.

The specific epithet chenhedongensis is derived from Chenhedong Provincial Nature Reserve, Conghua District, Guangzhou, Guangdong Province, China—the only locality from which the new species is currently known. The Chinese name is given as 陈禾洞黄芩 (chén hé dòng huáng qín).

##### Distribution and habitat.

To date, *Scutellaria chenhedongensis* is known only from its type locality at Chenhedong Provincial Nature Reserve, Conghua District, Guangzhou, Guangdong Province, China, where it grows along valleys at the edges of subtropical evergreen broad-leaved forests at an elevation of 444 m.

##### Additional specimens examined.

Digital images of the following specimens were examined through the Chinese Virtual Herbarium (CVH).

*Scutellaria subintegra*: China • Guangdong Province, in forest, 23 August 1931, W. Y. Chun 7116 (IBK00180330, type material).

*Scutellaria grossecrenata*: China • Guangdong Province, open hillside, 20 April 1934, X. P. Gao 54152 (PE00837255); • Guangdong Province, valley dense forest, 550 m, 13 April 2013, L. Wu & Y. Tong 3230 (BNU0029410).

*Scutellaria calcarata*: China • Yunnan Province, near Tanhua Temple, forest edge, 2650 m, 1 September 1963, Northwest Yunnan Expedition 6753 (KUN0353141).

*Scutellaria hunanensis*: China • Hunan Province, Huazhong University 10324 (PE00837268, holotype); • Hunan Province, 7 August 1953, K. J. Guan & B. M. Yang 00037 (PE00837267, paratype); • Hunan Province, 22 October 1953, 0437 (PE00837266, paratype).

*Scutellaria axilliflora*: China • Fujian Province, on rocks in shade, 933 m, 22 June 1925, X. X. Zhong 3545 (PE01690461, isotype).

##### Conservation status.

Field investigations conducted in this study confirm that *Scutellaria chenhedongensis* is currently known from a single locality within Chenhedong Provincial Nature Reserve, with a population of approximately 300 mature individuals. Given its highly restricted distribution and small population size, and in accordance with the IUCN Red List Categories and Criteria (IUCN 2024), the species is provisionally assessed as Vulnerable (VU D1+D2): the number of mature individuals is fewer than 1,000 (criterion D1), and the species is restricted to a single location (criterion D2), such that any potential local environmental disturbance could rapidly cause its status to deteriorate to Endangered or Critically Endangered within a short period.

Notably, the entire known population of *S. chenhedongensis* lies within the core area of Chenhedong Provincial Nature Reserve. The reserve, located in Lütian Town of Conghua District, Guangzhou, is the source area of the Liuxi River, and its vegetation is dominated by well-preserved subtropical evergreen broad-leaved forest. No conspicuous anthropogenic or natural threats to the population have been observed at present. The effective management of the reserve provides relatively favorable conditions for *in situ* conservation; nevertheless, given the highly restricted distribution of the species, systematic population monitoring is recommended, and a more precise determination of its extent of occurrence (EOO) and area of occupancy (AOO) is needed to refine the assessment of its threat status in subsequent evaluations.

## Supplementary Material

XML Treatment for Scutellaria chenhedongensis

## Additional information

### Artificial Intelligence (AI) use

The authors accept full responsibility for the content of the manuscript, including the disclosure of any use of AI.

Regarding the use of AI in the preparation of this manuscript, the authors declare the following: Claude.

**Used for**: Language, style and writing.

Claude was used to assist with translating the manuscript from Chinese to English, refining English language and style. All taxonomic descriptions, morphological observations, molecular phylogenetic analyses, and scientific conclusions are entirely the work of the authors.

### Author ORCIDs

X.-W. Yi https://orcid.org/0009-0004-8867-7418

Y.-F. Chen https://orcid.org/0009-0000-6191-2271

W. Xu https://orcid.org/0000-0003-2907-0478

P.-S. Zhong https://orcid.org/0009-0000-5915-4942

H.-J. Chen https://orcid.org/0009-0005-1045-5943

L.-R. Shen https://orcid.org/0009-0008-2746-3431

Q. Fan https://orcid.org/0000-0003-4254-6936

### Data availability

All of the data that support the findings of this study are available in the main text.
